# 2-(2,4-Dinitro­benz­yl)pyridinium 2-hy­droxy-3,5-dinitro­benzoate

**DOI:** 10.1107/S1600536810024888

**Published:** 2010-07-03

**Authors:** Graham Smith, Urs D. Wermuth, David J. Young

**Affiliations:** a Faculty of Science and Technology, Queensland University of Technology, GPO Box 2434, Brisbane, Queensland 4001, Australia; b School of Biomolecular and Physical Sciences, Griffith University, Nathan, Queensland 4111, Australia

## Abstract

In the structure of the title salt, C_12_H_10_N_3_O_4_^+^·C_7_H_3_N_2_O_7_^−^, the cations and the anions are linked by a single N^+^—H⋯O_carbox­yl_ hydrogen bond, the discrete cation–anion unit having no inter­molecular associations other than weak cation–anion aromatic ring π–π inter­actions [ring centroid separation = 3.7320 (14) Å] and a number of weak inter-unit aromatic C—H⋯O contacts. An intramolecular C—H⋯O hydrox­yl–carboxyl hydrogen bond occurs in the anion.

## Related literature

For structural data on 2-(2,4-dinitro­benz­yl)pyridine and related compounds, see: Seff & Trueblood (1968 ▶); Scherl *et al.* (1996 ▶); Naumov *et al.* (2002 ▶, 2005 ▶); Smith, Wermuth & Young (2010 ▶). For some structures of 3,5-dinitro­salicylic acid salts of Lewis bases, see: Smith *et al.* (2002 ▶, 2003 ▶, 2007 ▶); Smith, Cotton *et al.* (2010 ▶).
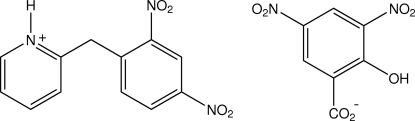


## Experimental

### 

#### Crystal data

C_12_H_10_N_3_O_4_^+^·C_7_H_3_N_2_O_7_^−^
*M* *_r_* = 487.34
Monoclinic, 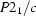

*a* = 7.1550 (2) Å
*b* = 21.7356 (5) Å
*c* = 13.2080 (4) Å
β = 104.424 (3)°
*V* = 1989.34 (10) Å^3^
*Z* = 4
Mo *K*α radiation
μ = 0.14 mm^−1^
*T* = 200 K
0.40 × 0.35 × 0.18 mm

#### Data collection

Oxford Diffraction Gemini-S CCD-detector diffractometer
Absorption correction: multi-scan (*SADABS*; Sheldrick, 1996 ▶) *T*_min_ = 0.960, *T*_max_ = 0.982
13759 measured reflections
3910 independent reflections
2865 reflections with *I* > 2σ(*I*)
*R*_int_ = 0.025

#### Refinement

*R*[*F* ^2^ > 2σ(*F* ^2^)] = 0.051
*wR*(*F* ^2^) = 0.153
*S* = 1.08
3910 reflections
320 parameters
H-atom parameters constrained
Δρ_max_ = 0.75 e Å^−3^
Δρ_min_ = −0.32 e Å^−3^

### 

Data collection: *CrysAlis PRO* (Oxford Diffraction, 2009 ▶); cell refinement: *CrysAlis PRO*; data reduction: *CrysAlis PRO*; program(s) used to solve structure: *SIR92* (Altomare *et al.*, 1994 ▶); program(s) used to refine structure: *SHELXL97* (Sheldrick, 2008 ▶); molecular graphics: *PLATON* (Spek, 2009 ▶); software used to prepare material for publication: *PLATON*.

## Supplementary Material

Crystal structure: contains datablocks global, I. DOI: 10.1107/S1600536810024888/ez2219sup1.cif

Structure factors: contains datablocks I. DOI: 10.1107/S1600536810024888/ez2219Isup2.hkl

Additional supplementary materials:  crystallographic information; 3D view; checkCIF report

## Figures and Tables

**Table 1:** Hydrogen-bond geometry (Å, °)

*D*—H⋯*A*	*D*—H	H⋯*A*	*D*⋯*A*	*D*—H⋯*A*
N1—H1⋯O11*A*	0.91	1.72	2.627 (3)	172
O2*A*—H2*A*⋯O12*A*	0.93	1.61	2.476 (3)	152
C3—H3⋯O11*A*^i^	0.93	2.48	3.246 (3)	140
C5—H5⋯O31*A*^ii^	0.93	2.56	3.051 (4)	114
C6—H6⋯O31*A*^ii^	0.93	2.48	3.020 (4)	117
C51—H51⋯O51*A*^iii^	0.93	2.55	3.137 (4)	122
C61—H61⋯O51*A*^iii^	0.93	2.54	3.141 (3)	123
C71—H72⋯O11*A*	0.97	2.55	3.247 (3)	129

Symmetry codes: (i) 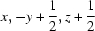; (ii) 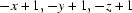; (iii) 
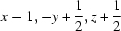
.

**Fig. 1.**
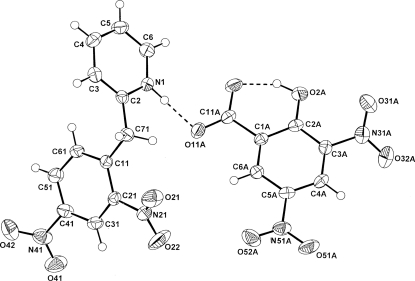
Molecular configuration and atom naming scheme for the DNBP cation and the DNSA anion in the asymmetric unit of (I), with the hydrogen bonds shown as a dashed lines. Displacement ellipsoids are drawn at the 50% probability level.

**Fig. 2.**
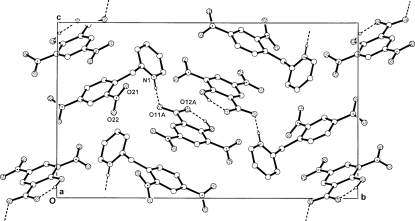
The packing of the cation–anion pairs in the unit cell viewed down the a cell direction. Non-interactive H atoms are omitted.

## supplementary crystallographic information

### Comment

The Lewis base 2-(2,4-dinitrobenzyl)pyridine (DNBP) is unusual because of its photochromic properties, undergoing a reversible solid-state colour change from colourless to deep blue on irradiation with light of wavelength 400 nm or less. This is due to two-photon excitation giving nitro-assisted proton transfer (NAPT) involving an oxygen of the *o*-nitro substituent group and the pyridine N atom (Naumov *et al.*, 2002). The structures of both the colourless form (Seff & Trueblood, 1968; Scherl *et al.*, 1996) and the blue form (Naumov *et al.*, 2002), have been determined as well, as that of the chloride (Naumov *et al.*, 2005).

Our reaction of DNBP with a number of aromatic carboxylic and sulfonic acids in aqueous ethanol has previously provided only one crystalline compound: bis[2-(2,4-dinitrobenzyl)pyridinium] biphenyl-4,4'-disulfonate trihydrate, for which the structure was reported (Smith, Wermuth & Young, 2010). A second crystalline compound was subsequently obtained from the reaction of DNBP with 3,5-dinitrosalicylic acid (DNSA), the title compound anhydrous C_12_H_10_N_3_O_4_^+^.C_7_H_3_N_2_O_7_^-^ (I), the structure of which is reported here. DNSA has been very useful as an acid capable of producing crystalline salts with a range of both aliphatic and aromatic Lewis bases and the structures of a large number of these are reported in the crystallographic literature, *e.g.* Smith *et al.* (2002, 2003, 2007) and Smith, Cotton *et al.* (2010).

With compound (I) (Fig. 1), a single cation–anion *N*^+^–*H*···O_carboxyl_ hydrogen bond together with a weak aliphatic *C*–*H*···O_carboxyl_ association (C71–H···O11A) (Table 1), form discrete cation–anion units. These units have no intermolecular interactions other than mostly weak aromatic C–H···O contacts [one is strong: C6–H···O31A^iii^: symmetry code (iii), -*x* + 1, -*y* + 1, -*z* + 1], and also weak cation–anion π–π aromatic ring associations [ring centroid separation for C11–C61 to C1A–C6A, 3.7320 (14) Å] down the *a* axial direction (Fig. 2).

With the DNBP cation both nitro groups are rotated out of the plane of the benzene ring [torsion angles C11–C21–N21–O22, -151.4 (2)° and C31–C41–N41–O42, 149.9 (2)°]. In the DNSA anion, within the intramolecular hydroxyl–carboxyl hydrogen bond, the H atom is located on the hydroxyl group rather than being *anti*-related on the carboxyl group. (I) is only one of the *ca* 30% of the known examples of DNSA salts having this (Smith *et al.*, 2007). The carboxyl group, as expected, is close to planar with the benzene ring [torsion angle C2A–C1A–C11A–O11A, 175.0 (2)°], while one nitro group is close to coplanar [torsion angle C4A–C5A–C51A–O52A, 177.6 (2)°], the other being rotated out of the plane [torsion angle C2A–C3A–C31A–O32A, -159.1 (2)°]. In the structure there is a short nonbonded contact across an inversion centre [O42···O42^iv^, 2.897 (3) Å: symmetry code (iv) -*x* + 1, -*y*, -*z* + 1], this O atom being associated with the large electron density maximum (0.75 e Å^-3^).

### Experimental

The title compound was synthesized by heating together under reflux for 10 minutes, 1 mmol quantities of 2-(2,4-dinitrobenzyl)pyridine and 3,5-dinitrosalicylic acid in 50 ml of 50% ethanol–water. After concentration to *ca* 30 ml, partial room temperature evaporation of the hot-filtered solution gave yellow plates (m.p. 383 K) having 90° yellow–colourless orientational dichroism in the crystals.

### Refinement

The two hydrogen atoms involved in hydrogen-bonding interactions were located by difference methods and were constrained in the refinement with the other H atoms at calculated positions [C–H = 0.93 Å (aromatic) and 0.97 Å (aliphatic)] and with *U*_iso_(H) = 1.2*U*_eq_(C), using a riding-model approximation. There is a larger than normal maximum residual electron density peak (0.75 e Å^-3^) which is located *ca* 1.56 Å from the nitro O atom O42).

### Crystal data

**Table:**

C_12_H_10_N_3_O_4_^+^·C_7_H_3_N_2_O_7_^−^	*F*(000) = 1000
*M**_r_* = 487.34	*D*_x_ = 1.627 Mg m^−^^3^
Monoclinic, *P*2_1_/*c*	Melting point: 383 K
Hall symbol: -P 2ybc	Mo *K*α radiation, λ = 0.71073 Å
*a* = 7.1550 (2) Å	Cell parameters from 6248 reflections
*b* = 21.7356 (5) Å	θ = 3.1–28.7°
*c* = 13.2080 (4) Å	µ = 0.14 mm^−^^1^
β = 104.424 (3)°	*T* = 200 K
*V* = 1989.34 (10) Å^3^	Plate, yellow
*Z* = 4	0.40 × 0.35 × 0.18 mm

### Data collection

**Table:**

Oxford Diffraction Gemini-S CCD-detector diffractometer	3910 independent reflections
Radiation source: Enhance (Mo) X-ray source	2865 reflections with *I* > 2σ(*I*)
graphite	*R*_int_ = 0.025
ω scans	θ_max_ = 26.0°, θ_min_ = 3.1°
Absorption correction: multi-scan (*SADABS*; Sheldrick, 1996)	*h* = −8→5
*T*_min_ = 0.960, *T*_max_ = 0.982	*k* = −26→26
13759 measured reflections	*l* = −16→16

### Refinement

**Table:**

Refinement on *F*^2^	Primary atom site location: structure-invariant direct methods
Least-squares matrix: full	Secondary atom site location: difference Fourier map
*R*[*F*^2^ > 2σ(*F*^2^)] = 0.051	Hydrogen site location: inferred from neighbouring sites
*wR*(*F*^2^) = 0.153	H-atom parameters constrained
*S* = 1.08	*w* = 1/[σ^2^(*F*_o_^2^) + (0.0819*P*)^2^ + 0.673*P*] where *P* = (*F*_o_^2^ + 2*F*_c_^2^)/3
3910 reflections	(Δ/σ)_max_ < 0.001
320 parameters	Δρ_max_ = 0.75 e Å^−^^3^
0 restraints	Δρ_min_ = −0.32 e Å^−^^3^

### Special details

**Table:**

Geometry. Bond distances, angles *etc*. have been calculated using the rounded fractional coordinates. All su's are estimated from the variances of the (full) variance-covariance matrix. The cell e.s.d.'s are taken into account in the estimation of distances, angles and torsion angles
Refinement. Refinement of *F*^2^ against ALL reflections. The weighted *R*-factor *wR* and goodness of fit *S* are based on *F*^2^, conventional *R*-factors *R* are based on *F*, with *F* set to zero for negative *F*^2^. The threshold expression of *F*^2^ > σ(*F*^2^) is used only for calculating *R*-factors(gt) *etc*. and is not relevant to the choice of reflections for refinement. *R*-factors based on *F*^2^ are statistically about twice as large as those based on *F*, and *R*- factors based on ALL data will be even larger.

### Fractional atomic coordinates and isotropic or equivalent isotropic displacement parameters (Å^2^)

**Table:**

	*x*	*y*	*z*	*U*_iso_*/*U*_eq_	
O21	1.2566 (3)	0.22969 (10)	0.63986 (18)	0.0562 (8)	
O22	1.1932 (4)	0.18930 (12)	0.48691 (19)	0.0733 (10)	
O41	0.7828 (4)	0.00564 (10)	0.43481 (18)	0.0635 (9)	
O42	0.6790 (3)	−0.02169 (9)	0.56819 (19)	0.0577 (8)	
N1	0.6649 (3)	0.32238 (8)	0.69232 (15)	0.0274 (6)	
N21	1.1587 (3)	0.19783 (10)	0.57118 (18)	0.0365 (7)	
N41	0.7462 (3)	0.01652 (11)	0.5177 (2)	0.0458 (8)	
C2	0.7755 (3)	0.28074 (10)	0.75516 (18)	0.0267 (7)	
C3	0.7361 (4)	0.26743 (11)	0.84941 (19)	0.0316 (7)	
C4	0.5867 (4)	0.29735 (12)	0.87819 (19)	0.0345 (8)	
C5	0.4801 (4)	0.34101 (12)	0.8133 (2)	0.0376 (8)	
C6	0.5212 (4)	0.35297 (12)	0.7190 (2)	0.0359 (8)	
C11	0.8856 (3)	0.19180 (10)	0.65949 (17)	0.0250 (7)	
C21	0.9877 (3)	0.16655 (10)	0.59134 (18)	0.0268 (7)	
C31	0.9395 (3)	0.11127 (11)	0.5400 (2)	0.0322 (8)	
C41	0.7909 (4)	0.07812 (11)	0.5632 (2)	0.0353 (8)	
C51	0.6845 (4)	0.10000 (12)	0.6292 (2)	0.0359 (8)	
C61	0.7296 (3)	0.15746 (11)	0.67349 (19)	0.0307 (7)	
C71	0.9381 (3)	0.25231 (10)	0.7169 (2)	0.0308 (7)	
O2A	0.7053 (3)	0.51546 (8)	0.39374 (15)	0.0389 (6)	
O11A	0.7728 (2)	0.34274 (8)	0.51916 (13)	0.0363 (6)	
O12A	0.6246 (3)	0.43376 (8)	0.50643 (15)	0.0404 (6)	
O31A	0.8926 (3)	0.60447 (8)	0.32114 (17)	0.0482 (7)	
O32A	1.0404 (4)	0.57457 (11)	0.20856 (19)	0.0703 (10)	
O51A	1.4121 (3)	0.38998 (10)	0.28140 (17)	0.0514 (7)	
O52A	1.3387 (3)	0.32069 (9)	0.38239 (19)	0.0536 (7)	
N31A	0.9667 (3)	0.56457 (10)	0.28107 (17)	0.0388 (7)	
N51A	1.3139 (3)	0.37048 (10)	0.33857 (18)	0.0375 (7)	
C1A	0.8825 (3)	0.42179 (10)	0.42470 (17)	0.0254 (6)	
C2A	0.8508 (3)	0.48139 (11)	0.38128 (18)	0.0276 (7)	
C3A	0.9808 (4)	0.50221 (10)	0.32341 (18)	0.0293 (7)	
C4A	1.1287 (3)	0.46614 (11)	0.30776 (18)	0.0296 (7)	
C5A	1.1555 (3)	0.40898 (11)	0.35306 (18)	0.0278 (7)	
C6A	1.0358 (3)	0.38671 (10)	0.41266 (18)	0.0268 (7)	
C11A	0.7494 (3)	0.39730 (11)	0.48798 (18)	0.0294 (7)	
H1	0.69100	0.32880	0.62900	0.0330*	
H3	0.80970	0.23840	0.89370	0.0380*	
H4	0.55830	0.28790	0.94140	0.0410*	
H5	0.38170	0.36210	0.83300	0.0450*	
H6	0.44980	0.38210	0.67380	0.0430*	
H31	1.00440	0.09700	0.49180	0.0390*	
H51	0.58490	0.07680	0.64350	0.0430*	
H61	0.65290	0.17380	0.71420	0.0370*	
H71	1.04700	0.24550	0.77630	0.0370*	
H72	0.97850	0.28120	0.67050	0.0370*	
H2A	0.64800	0.49380	0.43920	0.0470*	
H4A	1.20920	0.48020	0.26720	0.0350*	
H6A	1.05890	0.34830	0.44430	0.0320*	

### Atomic displacement parameters (Å^2^)

**Table:**

	*U*^11^	*U*^22^	*U*^33^	*U*^12^	*U*^13^	*U*^23^
O21	0.0376 (11)	0.0622 (13)	0.0758 (15)	−0.0202 (10)	0.0272 (11)	−0.0216 (12)
O22	0.0767 (17)	0.0939 (18)	0.0687 (15)	−0.0332 (14)	0.0546 (14)	−0.0196 (14)
O41	0.0775 (17)	0.0640 (14)	0.0585 (14)	−0.0200 (12)	0.0349 (13)	−0.0309 (12)
O42	0.0637 (14)	0.0356 (11)	0.0721 (15)	−0.0054 (10)	0.0135 (12)	0.0012 (11)
N1	0.0292 (10)	0.0257 (9)	0.0297 (10)	−0.0008 (8)	0.0120 (9)	0.0026 (8)
N21	0.0322 (12)	0.0356 (11)	0.0477 (13)	−0.0004 (10)	0.0212 (11)	0.0017 (11)
N41	0.0363 (13)	0.0420 (13)	0.0597 (16)	−0.0027 (10)	0.0132 (12)	−0.0135 (12)
C2	0.0264 (12)	0.0207 (10)	0.0338 (13)	−0.0042 (9)	0.0093 (10)	−0.0031 (10)
C3	0.0344 (13)	0.0284 (12)	0.0306 (13)	−0.0009 (10)	0.0057 (11)	0.0015 (10)
C4	0.0373 (14)	0.0405 (14)	0.0283 (13)	−0.0064 (11)	0.0133 (11)	−0.0037 (11)
C5	0.0367 (14)	0.0408 (14)	0.0391 (14)	0.0066 (12)	0.0164 (12)	−0.0033 (12)
C6	0.0341 (13)	0.0337 (13)	0.0412 (14)	0.0074 (11)	0.0121 (12)	0.0054 (11)
C11	0.0219 (11)	0.0259 (11)	0.0270 (12)	0.0036 (9)	0.0055 (9)	0.0055 (9)
C21	0.0223 (11)	0.0304 (12)	0.0289 (12)	0.0020 (9)	0.0087 (9)	0.0049 (10)
C31	0.0296 (13)	0.0357 (13)	0.0335 (13)	0.0058 (10)	0.0121 (11)	−0.0016 (11)
C41	0.0301 (13)	0.0322 (13)	0.0440 (15)	0.0014 (10)	0.0098 (11)	−0.0072 (12)
C51	0.0292 (13)	0.0363 (13)	0.0455 (15)	−0.0065 (11)	0.0153 (12)	−0.0050 (12)
C61	0.0277 (12)	0.0289 (12)	0.0393 (13)	0.0007 (10)	0.0158 (11)	−0.0019 (11)
C71	0.0284 (12)	0.0265 (12)	0.0396 (14)	−0.0002 (10)	0.0123 (11)	0.0002 (11)
O2A	0.0345 (10)	0.0364 (10)	0.0512 (11)	0.0054 (8)	0.0209 (9)	0.0012 (8)
O11A	0.0406 (10)	0.0372 (10)	0.0350 (9)	−0.0063 (8)	0.0169 (8)	0.0018 (8)
O12A	0.0391 (10)	0.0429 (10)	0.0475 (11)	−0.0027 (8)	0.0266 (9)	−0.0072 (9)
O31A	0.0479 (12)	0.0313 (10)	0.0636 (13)	0.0026 (9)	0.0108 (11)	0.0013 (9)
O32A	0.101 (2)	0.0591 (14)	0.0630 (15)	−0.0014 (13)	0.0434 (15)	0.0203 (12)
O51A	0.0386 (11)	0.0653 (13)	0.0604 (13)	0.0026 (9)	0.0311 (10)	−0.0076 (11)
O52A	0.0403 (11)	0.0374 (11)	0.0861 (16)	0.0093 (9)	0.0215 (11)	0.0020 (11)
N31A	0.0385 (12)	0.0389 (12)	0.0385 (12)	−0.0018 (10)	0.0087 (10)	0.0062 (10)
N51A	0.0249 (11)	0.0425 (13)	0.0475 (13)	0.0001 (9)	0.0133 (10)	−0.0094 (11)
C1A	0.0254 (11)	0.0286 (11)	0.0228 (11)	−0.0030 (9)	0.0073 (9)	−0.0043 (10)
C2A	0.0258 (12)	0.0325 (12)	0.0255 (12)	−0.0010 (10)	0.0082 (10)	−0.0051 (10)
C3A	0.0316 (13)	0.0290 (12)	0.0274 (12)	−0.0018 (10)	0.0074 (10)	0.0015 (10)
C4A	0.0262 (12)	0.0385 (13)	0.0265 (12)	−0.0069 (10)	0.0113 (10)	−0.0032 (11)
C5A	0.0226 (11)	0.0327 (12)	0.0288 (12)	−0.0007 (10)	0.0076 (10)	−0.0091 (10)
C6A	0.0265 (12)	0.0273 (12)	0.0266 (11)	−0.0024 (9)	0.0065 (10)	−0.0019 (10)
C11A	0.0285 (12)	0.0352 (13)	0.0264 (12)	−0.0073 (10)	0.0104 (10)	−0.0061 (10)

### Geometric parameters (Å, °)

**Table:**

O21—N21	1.215 (3)	C11—C61	1.393 (3)
O22—N21	1.213 (3)	C11—C71	1.518 (3)
O41—N41	1.211 (3)	C21—C31	1.381 (3)
O42—N41	1.235 (3)	C31—C41	1.381 (4)
O2A—C2A	1.321 (3)	C41—C51	1.377 (4)
O11A—C11A	1.253 (3)	C51—C61	1.383 (4)
O12A—C11A	1.262 (3)	C3—H3	0.9300
O31A—N31A	1.206 (3)	C4—H4	0.9300
O32A—N31A	1.222 (3)	C5—H5	0.9300
O51A—N51A	1.228 (3)	C6—H6	0.9300
O52A—N51A	1.219 (3)	C31—H31	0.9300
O2A—H2A	0.9300	C51—H51	0.9300
N1—C6	1.343 (4)	C61—H61	0.9300
N1—C2	1.344 (3)	C71—H71	0.9700
N21—C21	1.481 (3)	C71—H72	0.9700
N41—C41	1.470 (3)	C1A—C6A	1.377 (3)
N1—H1	0.9100	C1A—C11A	1.512 (3)
N31A—C3A	1.460 (3)	C1A—C2A	1.412 (3)
N51A—C5A	1.459 (3)	C2A—C3A	1.417 (4)
C2—C71	1.511 (3)	C3A—C4A	1.373 (4)
C2—C3	1.374 (3)	C4A—C5A	1.372 (3)
C3—C4	1.383 (4)	C5A—C6A	1.387 (3)
C4—C5	1.375 (4)	C4A—H4A	0.9300
C5—C6	1.374 (4)	C6A—H6A	0.9300
C11—C21	1.405 (3)		
			
C2A—O2A—H2A	106.00	C5—C4—H4	120.00
C2—N1—C6	122.9 (2)	C3—C4—H4	120.00
O21—N21—C21	118.4 (2)	C6—C5—H5	121.00
O22—N21—C21	117.5 (2)	C4—C5—H5	120.00
O21—N21—O22	124.1 (3)	N1—C6—H6	120.00
O41—N41—O42	123.9 (2)	C5—C6—H6	120.00
O42—N41—C41	117.8 (2)	C41—C31—H31	121.00
O41—N41—C41	118.3 (2)	C21—C31—H31	121.00
C6—N1—H1	121.00	C41—C51—H51	121.00
C2—N1—H1	116.00	C61—C51—H51	121.00
O31A—N31A—O32A	122.6 (2)	C11—C61—H61	119.00
O31A—N31A—C3A	119.7 (2)	C51—C61—H61	119.00
O32A—N31A—C3A	117.6 (2)	H71—C71—H72	108.00
O51A—N51A—C5A	117.7 (2)	C2—C71—H72	109.00
O52A—N51A—C5A	118.2 (2)	C11—C71—H71	109.00
O51A—N51A—O52A	124.1 (2)	C2—C71—H71	109.00
C3—C2—C71	124.5 (2)	C11—C71—H72	109.00
N1—C2—C3	118.6 (2)	C2A—C1A—C6A	120.9 (2)
N1—C2—C71	116.9 (2)	C2A—C1A—C11A	119.2 (2)
C2—C3—C4	119.8 (2)	C6A—C1A—C11A	119.9 (2)
C3—C4—C5	120.0 (2)	O2A—C2A—C3A	122.1 (2)
C4—C5—C6	119.0 (3)	C1A—C2A—C3A	116.8 (2)
N1—C6—C5	119.7 (2)	O2A—C2A—C1A	121.0 (2)
C61—C11—C71	120.3 (2)	N31A—C3A—C2A	120.7 (2)
C21—C11—C71	123.7 (2)	C2A—C3A—C4A	122.2 (2)
C21—C11—C61	116.0 (2)	N31A—C3A—C4A	117.0 (2)
N21—C21—C11	121.4 (2)	C3A—C4A—C5A	118.7 (2)
C11—C21—C31	123.3 (2)	N51A—C5A—C6A	118.9 (2)
N21—C21—C31	115.3 (2)	C4A—C5A—C6A	121.7 (2)
C21—C31—C41	117.2 (2)	N51A—C5A—C4A	119.4 (2)
N41—C41—C51	118.4 (2)	C1A—C6A—C5A	119.6 (2)
N41—C41—C31	119.1 (2)	O11A—C11A—C1A	117.7 (2)
C31—C41—C51	122.6 (2)	O12A—C11A—C1A	117.3 (2)
C41—C51—C61	118.2 (2)	O11A—C11A—O12A	125.0 (2)
C11—C61—C51	122.6 (2)	C3A—C4A—H4A	121.00
C2—C71—C11	113.96 (18)	C5A—C4A—H4A	121.00
C2—C3—H3	120.00	C1A—C6A—H6A	120.00
C4—C3—H3	120.00	C5A—C6A—H6A	120.00
			
C6—N1—C2—C3	2.1 (3)	C71—C11—C61—C51	−175.1 (2)
C6—N1—C2—C71	−177.0 (2)	C21—C11—C71—C2	160.2 (2)
C2—N1—C6—C5	−1.4 (4)	C61—C11—C71—C2	−20.4 (3)
O21—N21—C21—C11	29.4 (3)	C11—C21—C31—C41	−4.3 (4)
O21—N21—C21—C31	−149.7 (2)	N21—C21—C31—C41	174.8 (2)
O22—N21—C21—C11	−151.4 (2)	C21—C31—C41—N41	−174.5 (2)
O22—N21—C21—C31	29.5 (3)	C21—C31—C41—C51	4.2 (4)
O41—N41—C41—C31	−27.8 (4)	C31—C41—C51—C61	0.0 (4)
O41—N41—C41—C51	153.4 (3)	N41—C41—C51—C61	178.7 (2)
O42—N41—C41—C31	149.9 (2)	C41—C51—C61—C11	−4.5 (4)
O42—N41—C41—C51	−28.8 (4)	C6A—C1A—C2A—O2A	−179.2 (2)
O31A—N31A—C3A—C2A	24.4 (4)	C6A—C1A—C2A—C3A	1.4 (3)
O31A—N31A—C3A—C4A	−153.3 (2)	C11A—C1A—C2A—O2A	−1.1 (3)
O32A—N31A—C3A—C2A	−159.1 (2)	C11A—C1A—C2A—C3A	179.5 (2)
O32A—N31A—C3A—C4A	23.2 (3)	C2A—C1A—C6A—C5A	−3.0 (3)
O51A—N51A—C5A—C4A	−3.5 (3)	C11A—C1A—C6A—C5A	178.9 (2)
O51A—N51A—C5A—C6A	176.6 (2)	C2A—C1A—C11A—O11A	175.0 (2)
O52A—N51A—C5A—C4A	177.6 (2)	C2A—C1A—C11A—O12A	−5.8 (3)
O52A—N51A—C5A—C6A	−2.3 (3)	C6A—C1A—C11A—O11A	−6.9 (3)
C71—C2—C3—C4	178.3 (2)	C6A—C1A—C11A—O12A	172.3 (2)
N1—C2—C71—C11	−94.0 (2)	O2A—C2A—C3A—N31A	4.4 (4)
C3—C2—C71—C11	86.9 (3)	O2A—C2A—C3A—C4A	−178.0 (2)
N1—C2—C3—C4	−0.8 (4)	C1A—C2A—C3A—N31A	−176.2 (2)
C2—C3—C4—C5	−1.1 (4)	C1A—C2A—C3A—C4A	1.4 (3)
C3—C4—C5—C6	1.7 (4)	N31A—C3A—C4A—C5A	175.2 (2)
C4—C5—C6—N1	−0.5 (4)	C2A—C3A—C4A—C5A	−2.5 (4)
C61—C11—C21—N21	−178.8 (2)	C3A—C4A—C5A—N51A	−179.0 (2)
C61—C11—C21—C31	0.2 (3)	C3A—C4A—C5A—C6A	0.9 (4)
C71—C11—C21—N21	0.6 (3)	N51A—C5A—C6A—C1A	−178.2 (2)
C71—C11—C21—C31	179.6 (2)	C4A—C5A—C6A—C1A	1.9 (4)
C21—C11—C61—C51	4.3 (3)		

### Hydrogen-bond geometry (Å, °)

**Table:**

*D*—H···*A*	*D*—H	H···*A*	*D*···*A*	*D*—H···*A*
N1—H1···O11A	0.91	1.72	2.627 (3)	172
O2A—H2A···O12A	0.93	1.61	2.476 (3)	152
C3—H3···O11A^i^	0.93	2.48	3.246 (3)	140
C5—H5···O31A^ii^	0.93	2.56	3.051 (4)	114
C6—H6···O31A^ii^	0.93	2.48	3.020 (4)	117
C51—H51···O51A^iii^	0.93	2.55	3.137 (4)	122
C61—H61···O51A^iii^	0.93	2.54	3.141 (3)	123
C71—H72···O11A	0.97	2.55	3.247 (3)	129

Symmetry codes: (i) *x*, −*y*+1/2, *z*+1/2; (ii) −*x*+1, −*y*+1, −*z*+1; (iii) *x*−1, −*y*+1/2, *z*+1/2.
